# Bioactive Compounds from Microalgae and Cyanobacteria: Evaluation of Their Antioxidant and Antimicrobial Activities

**DOI:** 10.3390/md24050171

**Published:** 2026-05-09

**Authors:** Bruna de Falco, Carlos José Martel-Benítez, Carlos Almeida, Attilio Anzano, Francesco Pisapia, José Luis Martín-Barrasa, Antera Martel Quintana, Juan Luis Gómez-Pinchetti

**Affiliations:** 1 Banco Español de Algas, Fundación Parque Científico Tecnológico, Universidad de Las Palmas de Gran Canaria, Muelle de Taliarte s/n, 35214 Telde, Spain; calmeida@marinebiotechnology.org (C.A.); fpisapia@ufpcanarias.es (F.P.); 2 Department of Agricultural Sciences, University of Naples Federico II, Via Università 100, 80055 Portici, Italy; attilio.anzano@unina.it; 3 Fish Health and Infectious Diseases Group, University Institute of Animal Health and Food Safety (IUSA), University of Las Palmas de Gran Canaria, Carretera de Trasmontana s/n, 35416 Arucas, Spain; carlos.benitez@ulpgc.es (C.J.M.-B.); joseluis.martin@ulpgc.es (J.L.M.-B.); 4 Research Unit Hospital Universitario de Gran Canaria, Dr. Negrín, Fundación Instituto de Investigación Sanitaria de Canarias (FIISC), Bco. de la Ballena s/n, 35011 Las Palmas de Gran Canaria, Spain; 5 Research Unit Hospital Universitario de Gran Canaria, Dr. Negrín, Instituto de Investigación Sanitaria de Canarias (IISC), Eukaryotic-Prokaryotic Synergy-Comparative and Translational Medicine Group (Cardiorespiratory Infectious Diseases and Epidemiology Area), Bco. de la Ballena s/n, 35019 Las Palmas de Gran Canaria, Spain; 6 Faculty of Health Sciences, University Fernando Pessoa-Canarias, C/la Juventud s/n, 35450 Santa María de Guía, Spain; 7 CIBER de Enfermedades Infecciosas (CIBERINFEC), Instituto de Salud Carlos III, 28029 Madrid, Spain; 8 Banco Español de Algas, Instituto de Oceanografía y Cambio Global (IOCAG), Universidad de Las Palmas de Gran Canaria, 35214 Telde, Spain; antera.martel@ulpgc.es (A.M.Q.); juan.gomez@ulpgc.es (J.L.G.-P.)

**Keywords:** biodiversity, gas chromatography, fatty acids, pigments, ultrasonic assisted extraction, antibacterial activity, *Staphylococcus aureus*, cyanobacteria, microalgae

## Abstract

Microalgae hold great potential towards pharmaceutical and nutraceutical sectors due to their substantial content of highly functional bioactive compounds. To assess their potential as a sustainable source of valuable products, 10 cyanobacterial and 10 eukaryotic microalgal strains belonging to different taxonomic groups (Chlorophyta, Cyanophyta, Euglenophyta, Heterokontophyta and Rhodophyta) were screened for their biochemical profile, antioxidant and antimicrobial activity against *Staphylococcus aureus*. Total phenol content and antioxidant activity were positively correlated (r = 0.69, *p* < 0.01), with the highest values observed in *Euglena cantabrica*, *Haematococcus pluvialis*, and *Chrysoreinhardia giraudii*. HPLC-PAD pigment analysis revealed species-specific profiles, with β,β-carotene as major carotenoid in most cyanobacteria, whereas neoxanthin, violaxanthin and lutein were predominantly present in Chlorophyta, and fucoxanthin was the main carotenoid in *C. giraudii*, *Entomoneis* sp. and *Isochrysis galbana*. Protein content ranged from 9.2 ± 0.4% to 57.6 ± 0.5% with the highest levels in the cyanobacteria *Microcystis aeruginosa*, *Nostoc* sp., *Cylindrospermum stagnale*, *Anabaena minutissima*, and *Arthrospira platensis*. Multivariate analysis differentiated cyanobacteria and eukaryotes based on their fatty acid profiles. Organic extracts from 15 species showed inhibitory effects against *S. aureus* with MIC50 < 1024 µg/mL. The eukaryotes *Entomoneis* sp., *C. giraudii*, *I. galbana*, *Picochlorum* sp. and the cyanobacteria *C. stagnale* and *Nodularia* sp. exhibited the strongest inhibitory effects on bacterial growth. In conclusion, *E. cantabrica* and *C. giraudii* stood out for their high antioxidant activity and significant antimicrobial effects, respectively, highlighting their potential as valuable sources of bioactive compounds.

## 1. Introduction

Microalgae, which include both eukaryotic microorganisms and prokaryotic cyanobacteria (often referred to as blue-green algae), have emerged as valuable resources in biotechnology and applied sciences due to their exceptional capacity for synthesizing diverse bioactive compounds. These photosynthetic organisms thrive across a vast array of aquatic ecosystems and can produce a rich spectrum of metabolites with significant biological activities, including structural polysaccharides, essential vitamins, complex carbohydrates, high-value proteins, diverse lipid profiles, unique pigments, polyunsaturated fatty acids (PUFAs), and phenolic compounds [1]. The metabolic versatility of microalgae not only underscores their role in ecological resilience but also positions them as sustainable sources for novel compounds with applications in nutraceuticals, pharmaceuticals, and environmental remediation. Given their potential for high-yield cultivation and adaptability to various environmental conditions, microalgae are increasingly recognized as a cornerstone in the development of bio-based industries.

In recent decades, microalgae have established as a promising biomass source for a wide range of bioproducts, including biofuels [2]. These emerging non-edible oil sources, along with seaweed, have become key components of third-generation biodiesel feedstocks [3]. Microalgae’s rapid growth rates, high lipid content, and ability to adapt to wide range of environments, including wastewater and saline waters, give them significant competitive advantages. These traits allow microalgae cultivation to avoid the use of arable land and freshwater resources, making them a highly attractive option for biofuel production. Thus, biofuel production from microalgae offers a sustainable and renewable energy source that can contribute to reducing overall CO_2_ emissions and decreasing dependence on fossil fuels. Moreover, as consumer preferences have shifted towards healthier dietary choices, the demand for microalgae as food sources has expanded in Western countries. This growing recognition of microalgae as “novel food” has driven the development of innovative algal-derived products [4]. Particularly, their high content of carotenoids, phycobiliproteins, and PUFAs offers avenues for the development of novel nutraceuticals and functional ingredients [5]. Microalgae and cyanobacteria have also emerged as promising candidates for various medical applications. Indeed, the bioactive compounds derived from these microorganisms have shown substantial potential in the field of medicine, particularly in the development of novel therapeutics [6]. Recent studies have demonstrated their antioxidant properties, which can effectively mitigate oxidative stress, a key factor in the pathogenesis of diseases such as cardiovascular disorders and neurodegenerative conditions [7]. Additionally, bioactive compounds derived from microalgae and cyanobacteria have shown notable antimicrobial activity, offering novel therapeutic options in the fight against microbial infections, including those induced by drug-resistant pathogens [8]. Microalgae have also been explored for their anti-inflammatory, immunomodulatory, and anti-cancer properties [9]. Although microalgae have gained significant recognition as rich sources of numerous beneficial compounds and hold high potential for human nutrition, the European Commission has granted authorization for the consumption and use of only a limited number of species [10]. This is particularly striking considering the growing body of evidence highlighting the innovative and beneficial effects of microalgae-derived compounds currently used for applications in human nutrition, pharmaceuticals, and cosmetics [11]. Despite the promising developments, there appears to be an under-investment by relevant market operators in exploring and harnessing the full potential of these microorganisms.

The aim of the present study is to provide further evidence of the high potential of these microorganisms including species that have not yet been explored. This research evaluates the nutrient profile of 10 eukaryotic microalgal and 10 cyanobacterial strains. The strains are native to the Macaronesian bioregion, in particular from the Cape Verde and Canary Islands archipelagos, except for *Nostoc* sp. (BEA 0864B) from Moroccan, *Synechocystis* sp. (BEA 1833B) from mainland Spain, and *A. platensis* (BEA 0007B) from Lake Chad (Chad). The study includes the determination of total phenols, chlorophylls, and carotenoids, as well as the content of lipids, proteins, and the fatty acid profiles. Additionally, relevant bioactivity studies, including antioxidant and antimicrobial activities, are also presented.

## 2. Results and Discussion

### 2.1. Culture Growth Characteristics

Both groups of microalgae (Table 1), eukaryotes and cyanobacteria, exhibited growth rates ranging from 0.2 to 0.5 day^−1^, with the exception of *E. cantabrica* (0.1 day^−1^), which was in the “palmella” phase—a stage of euglenoids where they metabolize the polysaccharide β-glucan and do not exhibit active growth. Among the production values, *C. giraudii* (0.159 g CDW/L/d) and *Rhodosorus marinus* (0.112 g CDW/L/d) showed the highest rates. The remaining production values ranged from 0.013 to 0.064 g CDW/L/d (Table 1).

### 2.2. Total Phenol Content and Antioxidant Activity

**Figure 1:**
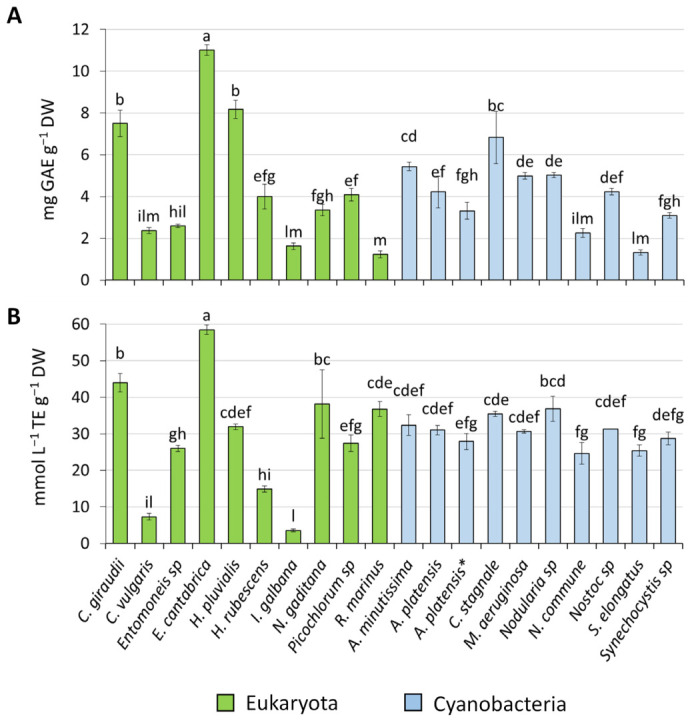
Total phenol content (**A**) and antioxidant activity (**B**) of 10 eukaryotic microalgae and 10 cyanobacterial strains. Different case letters indicate significant differences (*p* < 0.05) as determined by the post hoc Tukey’s test. *A. platensis* refers to BEA 0007B strain, A. platensis* refers to BEA 1257B strain.

The total phenol content (TPC) and antioxidant activity (AA) of ethanolic extracts from the selected microalgae results are presented in Figure 1. TPC ranged from 1.23 to 11.01 mg GAE g^−1^ of DW, with the lowest value found in *R. marinus*. The highest phenolic content was obtained in *E. cantabrica*, followed by *H. pluvialis* and *C. giraudii* with 8.18 ± 0.44 and 7.50 ± 0.63 mg GAE g^−1^ DW, respectively. The current results are consistent with previous studies, which also reported that *E. cantabrica* contained high concentration of phenolic compounds, specifically gallic acid and protocatechuic acid (5.87 and 2.97 mg⋅g^−1^ DW, respectively) [12,13]. The phenolic content of the strains *A. platensis* and *C. vulgaris* in the present study is similar to that described by Matos et al. [14]. However, our data reveal higher values in *N. commune* and *A. minutissima* compared to the findings reported by Vega et al. [15]. Additionally, their rapid growth rate value (0.47 day^−1^) suggests that *N. commune* and *A. minutissima* are excellent sources for specific metabolite production and hold promising implications for the scalability of the culture system.

As for TPC, *E. cantabrica* showed the highest AA (58.49 ± 1.24 mmol L^−1^ TE g^−1^ DW) followed by *C. giraudii* (43.97 ± 2.47 mmol L^−1^ TE g^−1^ DW), whereas the lowest AA was found in *I. galbana* with 3.56 ± 0.40 mmol L^−1^ TE g^−1^ DW.

Spearman correlation coefficients between AA and TPC demonstrated a statistically significant and robust correlation, with r = 0.69 (*p* < 0.01). A moderate correlation was observed between AA and chlorophyll-*a* (r = 0.426, *p* < 0.01), while the correlation between the antioxidant capacity and carotenoid content was weak (r = 0.262, *p* < 0.05). Although previous studies reported an insignificant correlation between AA and TPC or pigment content [16,17], our results closely align with those of Goiris et al. [18], suggesting that polyphenols might be major contributors to the antioxidant capacities of the selected microalgae.

### 2.3. Pigment Analysis

**Figure 2:**
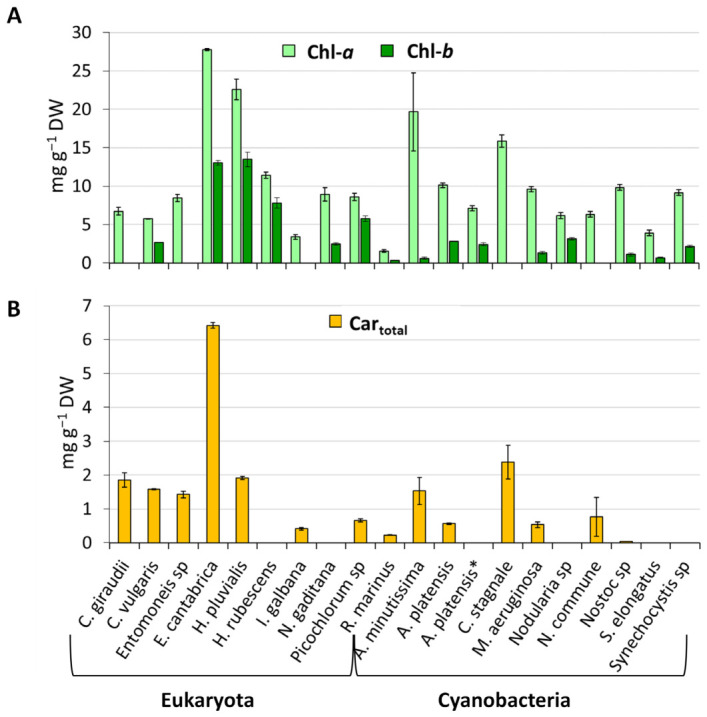
Chlorophyll-*a*, chlorophyll-*b* (**A**) and total carotenoids (**B**) content from ethanol extracts of 10 eukaryotic microalgae and 10 cyanobacteria. *A. platensis* refers to the species with code BEA 0007B, A. platensis* refers to the species with code BEA 1257B.

Significant variations in chlorophylls and carotenoids content among the analyzed species clearly indicate a species-specific pigment profile Figure 2 shows the spectrophotometric analysis results. Chlorophyll-*a* was the predominant pigment across all microalgae, which is an expected outcome due to its essential role in the photosynthetic process [19]. Its content ranged from 1.56 ± 0.15 mg g^−1^ DW in *R. marinus* to 27.76 ± 0.13 mg g^−1^ DW in *E. cantabrica*. Notably, *E. cantabrica* also exhibited the highest content of total carotenoids with a value of 6.42 ± 0.08 mg g^−1^ DW.

For a more comprehensive exploration of lipophilic pigments, fresh algal cultures were examined to analyze chlorophyll and carotenoids profiles by HPLC-PAD. The identified pigments, along with their retention time and the UV-visible data are reported in Table 2.

A direct quantitative comparison between data obtained by the spectrophotometric method with data obtained by HPLC-PAD is not recommended due to methodological differences related to different extraction processes and different analytical techniques used for the analysis. The freeze-drying process, when compared to fresh algal culture, can lead to chemical changes and pigment degradation. On the other hand, enzymatic reactions can still occur in fresh cultures, potentially resulting in the conversion of pigments into their respective derivatives.

Fucoxanthin and lutein were quantified using external calibration curves based on analytical standards. Results are reported only for the species in which these metabolites were detected (Table 3).

Microalgae typically exhibit fucoxanthin content ranging from 1 to 10 mg g^−1^ DW, with certain species accumulating over 20 mg g^−1^ of this pigment [19]. In our study, fucoxanthin emerged as the principal carotenoid in *C. giraudii* and *Entomoneis* sp. (Figure S2 of Supplementary Material). Particularly, *Entomoneis* sp. showed the highest content of this pigment, followed by *C. giraudii* and *I. galbana* (Table 3). Although data on fucoxanthin content in *C. giraudii* and *Entomoneis* sp. are scarce in the scientific literature, several studies reported values of fucoxanthin in *I. galbana* varying from 9 to 18 mg g^−1^ DW [20,21]. Our findings, however, showed lower values, aligning more with findings reported by other authors (1.88 mg g^−1^ DW and 2.19 ± 0.02 mg g^−1^ DW) [22,23]. This difference may be attributed to several factors such as environmental stress and culture conditions, including temperature, salinity, irradiance, and nutrient concentration. Fucoxanthin has attracted significant interest across multiple industrial sectors due to its diverse biological activities, among them anticancer, anti-obesity, antioxidant, antidiabetic and anti-inflammatory properties [24]. Nevertheless, its production remains limited due to the challenges in extraction efficiency from marine organisms, gaps in understanding its biosynthesis pathway and limited assessment of large-scale outdoor cultivation [19]. Our data suggests that species within the diatom genus *Entomoneis* could be a promising source of fucoxanthin. Further studies are needed to improve its growth rate and mass productivity to explore the feasibility of commercial production.

The strains belonging to Chlorophyta division (*C. vulgaris*, *H. pluvialis*, *Halochlorella rubescens* and *Picochlorum* sp.) were rich in neoxanthin, violaxanthin, and lutein (Figure S2, Supplementary Material), in accordance with previous studies [25]. Regarding the absolute quantification of lutein, *H. pluvialis* showed the highest content of this pigment, followed by *Picochlorum* sp. and *C. vulgaris* (Table 3). In this study, lutein content in *Nannochloropsis gaditana* fell below the limit of quantification (LOQ), whereas previous research reported 0.21 mg g^−1^ DW [26].

Although lutein content in *H. rubescens* could not be quantified (value below LOQ), relative quantification indicates its prominence as the main carotenoid, similar to that observed in *C. vulgaris* and *Picochlorum* sp. (Figure S2 of Supplementary Material), data in agreement with the existing literature [27,28,29]. Diadinoxanthin emerged as a characteristic pigment of euglenophytes, haptophytes and diatoms in our data, an outcome consistent with previous findings in the literature, which have reported the abundance of this pigment across these divisions [30,31,32].

Within cyanobacteria, β,β-carotene is the most abundant carotenoid except in *S. elongatus* where our data revealed xanthophylls as the major component of carotenoids, including zeaxanthin, synechoxanthin and caloxanthin. Previous studies have also reported these pigments as the most abundant carotenoids in *Synechococcus* [33,34,35,36]. Moreover, zeaxanthin was another dominant carotenoid in most cyanobacteria and in the rhodophyte *R. marinus* (Figures S2 and S3 of Supplementary Material).

### 2.4. Total Lipids and Fatty Acids Profile

The total lipid content (TL) ranged from 4.3 to 20.2% of the freeze-dried algal biomass (Table 4). Our TL data for *C. vulgaris*, *N. gaditana*, and *H. pluvialis* align with the existing literature, which has widely explored these species for biodiesel production due to their fast growth rate, high lipid content, and ability to thrive in various environments [37,38,39]. The highest lipid content was observed in *E. cantabrica* (20.2%) followed by *H. pluvialis* (18.9%), *N. gaditana* (18.9%), and *C. vulgaris* (18.5%) (Table 4). These strains show promising potential for biofuel production because of their high lipid content. However, despite the high lipid yield, *E. cantabrica* exhibited a relatively low growth rate, recorded at 0.1 day^−1^ and its biomass productivity was 0.049 gCDW·l^−1^·d^−1^ (Table 1). Therefore, further optimization is needed to enhance the growth rate and overall biomass productivity of *E. cantabrica*.

Strategies such as nutrient optimization, genetic modification, and controlled cultivation conditions (e.g., light intensity, temperature, and CO_2_ supply) have been explored in the literature to improve the productivity of microalgal species for biofuel purposes. For instance, studies on microalgae like *Nannochloropsis* and *Chlorella* species have shown that manipulating nitrogen and carbon supplies can lead to higher lipid accumulation and growth rates [40,41], suggesting that similar approaches could be employed for *E. cantabrica*, given the scarcity of the existing literature concerning this species.

The percentage of total saturated fatty acids (SFAs), monounsaturated fatty acids (MUFAs), and polyunsaturated fatty acids (PUFAs) is reported in Table 4. Among the studied species, *Entomoneis* sp. showed the highest amount of MUFAs (34.1%), with palmitoleic acid (C16:1n7) accounting for 26.1% (Table S1, Supplementary Material). Palmitoleic acid was also found to be particularly abundant in *N. gaditana* (27.2 ± 0.46%) and *Synechocystis* sp. (27.3 ± 0.14%), which is consistent with earlier research [29].

*E. cantabrica* exhibited the highest amounts of PUFAs, accounting for 55.1% of the total FAs and an n6:n3 ratio of 1.6. These findings underscore the potential of this species as an exceptional source for the nutraceutical industry. The traditional human diet, before the advent of processed foods and modern agricultural practices, is estimated to have had a ratio of omega-6 to omega-3 fatty acids that was close to 1:1 or 2:1. However, in many Western diets today, the ratio is much higher, often exceeding 10:1 or even 20:1. An excess of omega-6 PUFAs and a high n6:n3 ratio contributes to the development of several diseases, such as cardiovascular diseases, cancer, as well as inflammatory and autoimmune disorders [42]. Consequently, the recommended n6:n3 ratio is closer to 1 or at least lower than 4:1. In the current study, all analyzed species, except for the cyanobacteria *A. platensis* and *S. elongatus*, exhibited a n6:n3 ratio lower than 4, and in most cases, lower than 1 (Table 4). Among the species with the n6:n3 ≤ 1, only *H. rubescens*, *H. pluvialis*, and *Picochlorum* sp. showed low contents of SFAs (≤25%); hence, these microalgae could be a very good source for meeting the PUFA requirements in a well-balanced human diet.

The fatty acid profiles of the studied microalgae are shown in Table S1 of Supplementary Material. Palmitic acid (C16:0) was the most abundant fatty acid found in all microalgae species, accounting for approximately the half of the total FAs in *M. aeruginosa* and *R. marinus* (50.3 ± 0.2 and 46.5 ± 0.25%, respectively). Within the PUFAs, linoleic acid (C18:2n6) and α-linolenic acid (C18:3n3) were the most common FAs present in all microalgae, except for *Entomoneis* sp., *N. gaditana* and *Synechocystis* sp. In these strains the content of the eicosapentaenoic acid (EPA, C20:5n3) was particularly abundant with values of 6.9%, 18%, and 18.6%, respectively. EPA and docosahexaenoic acid (DHA) are two essential omega-3 PUFAs that play a critical role in human health. These long-chain fatty acids are well known for their cardiovascular benefits, including reducing triglyceride levels, lowering blood pressure, and decreasing inflammation [43]. Additionally, they are essential for brain function, contributing to neurological development and cognitive health [43]. Due to their anti-inflammatory properties, EPA and DHA are also associated with the prevention and management of chronic conditions such as arthritis and other inflammatory disorders [43]. Given their wide range of health benefits, the World Health Organization recommends a daily intake of at least 250 mg of combined EPA and DHA for adults [44]. Considering the importance of these omega-3 PUFAs, optimizing their content in microalgae species could enhance their nutraceutical value. Previous studies have shown that EPA levels in *N. gaditana* can increase significantly (from 19.13 ± 0.08% to 37.83 ± 0.37%) under varying salinity, light intensity, and photoperiod conditions [39]. This indicates that exploring different growth conditions could be a promising approach to enhance EPA levels in other species as well, potentially increasing their suitability for nutraceutical and biofuel applications. In agreement with previous reports, *I. galbana* was found to have a relatively high content of DHA (C22:6 n3), accounting for 3.2% of total FAs [26].

**Figure 3:**
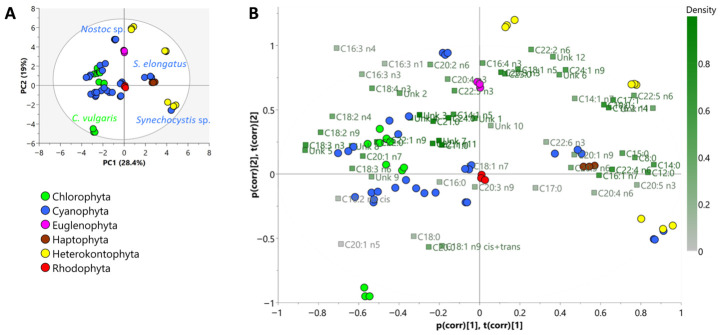
PCA score plot showing the first and second principal components (**A**) and bi-plot (score/loading plot) of all fatty acids (**B**). Scores are indicated as circles and loadings are indicated as boxes.

To model the differences between classes, the fatty acid profiles of each species were submitted to unsupervised principal component analysis (PCA) and supervised orthogonal partial least squares-discriminant analysis (OPLS-DA). The PCA plot shown in Figure 3A separated the different taxonomical groups (Chlorophyta, Cyanophyta, Euglenophyta, Haptophyta, Heterokontophyta and Rhodophyta) based on their fatty acid profiles. The resulting PCA score plot explains 47.4% of the total variance, with component 1 accounting for 28.4% and component 2 accounting for 19% (*p* < 0.05 for both components).

Specifically, in the PCA plot, Chlorophyta and Heterokontophyta were separated into two opposing quadrants, while Rhodophyta was precisely in the center of the plot. This positioning suggests an intermediate fatty acid profile of *R. marinus* among all the analyzed species. Most members of the Cyanophyta group formed a distinct cluster, with the exceptions of *Nostoc* sp., *S. elongatus*, and *Synechocystis* sp. Similarly, *C. vulgaris* was slightly separated from other Chlorophyta along the second component, likely due to its high levels of C18:0 and C20:0, as indicated by the loadings in the bi-plot (Figure 3B). Fatty acid variables such as C16:0, C18:0, C18:2n6, and C18:3n3 could serve as chemotaxonomic biomarkers for both Chlorophyta and Cyanophyta, with the exception of *Synechocystis* and *S. elongatus*, which separated from other cyanophytes along the first component probably due to their low level of C18:3n3, which is positioned on the opposite side of the bi-plot relative to these two strains (Figure 3B). Meanwhile, C16:1n7 could be a potential biomarker for both Heterokontophyta and Cyanophyta members. However, more comprehensive studies are needed to clearly define the fatty acids that can reliably serve as biomarkers across different divisions or species.

**Figure 4:**
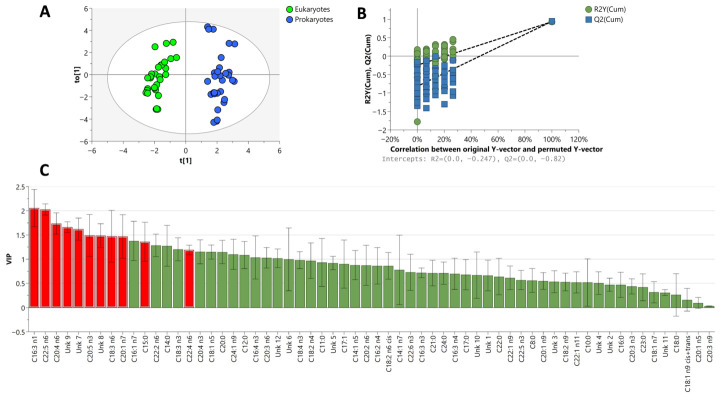
OPLS-DA model scores plot (**A**), permutation test (**B**), and VIP plot (**C**). In the VIP plot the discriminative fatty acids variables are highlighted in red (VIP ≥ 1 and *p*-value < 0.05).

Furthermore, OPLS-DA was performed to assess the relative contribution of the different variables to discriminate between eukaryotes and prokaryotes groups (Figure 4A). The OPLS-DA showed a distinct clustering in the fatty acid profiles, a good predictive ability value (Q^2^ = 0.94), good fitness of model value (R^2^Y = 0.93), and passed the validity permutation test (Figure 4B). These values indicate that the models are less likely to be overfitted as they are higher than the recommended values of 0.50 of R^2^X, R^2^Y and Q^2^ for a robust model [45].

**Figure 5:**
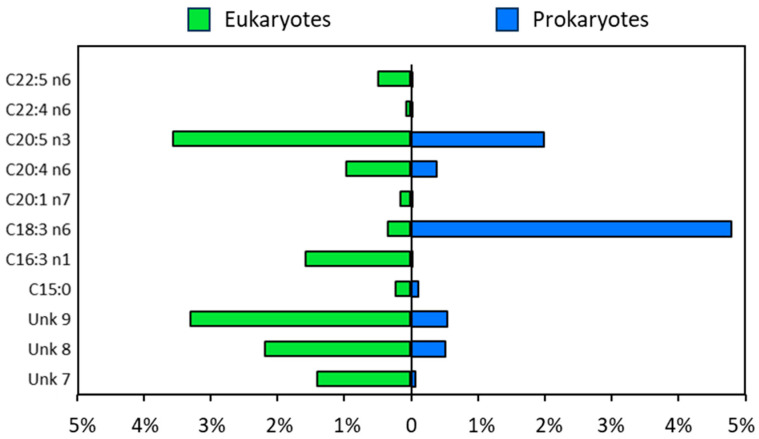
Relative levels expressed as a percentage (%) of significantly altered fatty acids in the eukaryotes compared to prokaryotes. Unidentified peaks are annotated as unknown (Unk).

Fatty acids with a variable importance in the projection (VIP) score ≥ 1 and *p*-value < 0.05 are considered discriminative for the separation between two groups in the OPLS-DA model and they were highlighted in red in the corresponding VIP plot (Figure 4C). The level of γ-linolenic acid (C18:3n6) was found to be high in the samples of prokaryotes group compared to eukaryotes (Figure 5), especially in *A. platensis* and *M. aeruginosa* strains (Table S1 of Supplementary Material).

Interestingly, C16:3n1 fatty acid, which has demonstrated antimicrobial properties, was found to be more abundant in eukaryotic species compared to prokaryotic ones. This is notable, as C16:3n1 is a relatively rare fatty acid in nature, with limited records of its occurrence. Previous reports have identified structurally related unsaturated fatty acids in natural sources, including marine diatoms, where they exhibit selective antibacterial activity against *S. aureus* [46]. The higher abundance of C16:3n1 in eukaryotes could indicate a broader role in cellular metabolism and defense mechanisms against microbial pathogens. Further analysis might be interesting to identify the unknown compounds, especially the unknown 7, 8, and 9, which appeared to be higher in eukaryotic species (Figure 5).

### 2.5. Protein Analysis

The total proteins of the selected microalgae are presented in Table 4. Overall, cyanobacteria exhibited a higher protein content compared to eukaryotes. This difference could likely be attributed to the presence of phycobiliproteins, mainly found in cyanobacteria. The highest values were observed in *A. minutissima*, *C. stagnale, M. aeruginosa*, and *Nostoc* sp., all of which surprisingly exhibited higher values than both *A. platensis* species. Microalgae are considered alternative protein sources and are used as food supplements for both animal and human nutrition. The exploration of microalgae as protein sources is primarily focused on cyanobacteria, with particular attention to *A. platensis,* as it is one of the richest sources of proteins of microbial origin (46–63% dry biomass) [47]. Our findings suggest that, beyond *A. platensis* other microalgae might also be a promising non-traditional source of proteins for human and animal consumption.

### 2.6. Antibacterial Activity

MIC50 and MIC90 values of the different organic extracts are summarized in Table 5, while data for each of the twenty *S. aureus* strains tested are presented in Table S2 of the Supplementary Material. *Entomoneis* sp. (MIC50 = 32 µg/mL, MIC90 = 32 µg/mL), *C. giraudii*, (MIC50 = 128 µg/mL, MIC90 = 128 µg/mL), *I. galbana* (MIC50 = 128 µg/mL, MIC90 = 256 µg/mL), and *Picochlorum* sp. (MIC50 = 128 µg/mL, MIC90 = 256 µg/mL) had the strongest inhibitory effects on bacterial growth among the eukaryote group. In the cyanobacteria group, *C. stagnale* (MIC50 = 128 µg/mL, MIC90 = 256 µg/mL), and *Nodularia* sp. (MIC50 = 128 µg/mL, MIC90 = 512 µg/mL) showed the best inhibitory effects on bacterial growth. Nevertheless, none of the *S. aureus* strains tested were susceptible to any aqueous extracts, with MICs > 1024 µg/mL. Although fatty acids are known to exhibit antimicrobial activity, particularly against Gram-positive bacteria, in our study no significant correlation was observed between antibacterial activity and total lipid content or fatty acid composition, including MUFA and PUFA fractions. Free fatty acids and monoglycerides have been reported to inhibit microbial growth, with activity depending on both the degree of unsaturation and carbon chain length [48,49]. However, the lack of correlation observed in our dataset suggests that fatty acids are not the primary contributors to the antimicrobial activity of the apolar fractions analyzed. This indicates that other non-polar bioactive metabolites, not specifically investigated in the present study, are likely responsible for the observed antibacterial effects.

## 3. Materials and Methods

### 3.1. Materials

Chemical solvents for analysis such as acetone, methanol, acetonitrile, and hexane were all HPLC and GC grade obtained by BioSigma (Santa Cruz de Tenerife, Spain). Other reagents including chloroform, dichloromethane, toluene, diethyl ether, sodium carbonate, potassium bicarbonate and ammonium sulphate were all ACS grade and from the Panreac brand (Barcelona, Spain). Analytical standards of fatty acids methyl esters (CRM47885 Supelco 37 Component), the internal standard nonadecanoic acid, the radical 1,1-diphenyl-2-picrylhydrazyl (DPPH), 6-hydroxy-2,5,7,8-tetramethyl-chroman-2-carboxylic acid (Trolox), gallic acid, Folin reagent, butylated hydroxytoluene (BHT), standard of fucoxanthin, lutein, and acetanilide were acquired from Sigma-Aldrich Chemie (Steinheim, Germany). The mixture of phytoplankton pigments used as the standard for pigment identification was obtained by DHI Laboratory Products (Hørsholm, Denmark).

### 3.2. Species Selection, Culture Conditions and Biomass Harvesting

Microalgae and cyanobacteria were selected based on four primary considerations: (1) robustness: robust strains exhibit resilience to various environmental stressors, including fluctuations in temperature, pH, salinity, and nutrient availability; (2) growth rate: although growth rate should be balanced with other factors such as biomass quality, a high growth rate can significantly increase biomass production; (3) ease of harvesting: harvesting can be a time-consuming and costly process and (4) biotechnological potential: some strains produce high-value bioproducts like lipids, pigments, or bioactive compounds that can be used in other biotechnological processes following the biorefinery concept. All clonal strains (Table 1) were provided by the Culture Collection of the Banco Español de Algas (BEA) in Gran Canaria, Spain. The microalgae were cultured in triplicate in 2 L Erlenmeyer flasks using appropriate culture media (see below), under controlled conditions with a light intensity of 120.0 ± 10 µmol photon/m^2^/s and a temperature of 24 ± 1 °C. Illumination was provided by a linear LED fluorescent lamp (model P/TUBE120/18/F/M/T8/CW) emitting a cool white spectrum with a correlated color temperature of 6500 K. Photon flux density (PFD) at the sample level was measured using a quantum sensor (LI 1400, Li-Cor Biosciences, Lincoln, NE, USA) equipped with a spherical quantum sensor (SPQA 2770), and expressed as photosynthetic photon flux density (PPFD, μmol m^−2^ s^−1^). Measurements were taken at the level of the experimental units under steady-state operating conditions to ensure uniform light distribution and reproducibility. Cultures were maintained on a 16:8 light:dark cycle and were aerated (bubbled) with continuous filtered air (0.2 µm glass filter) and CO_2_ pulses lasting 15 s/h only during daylight hours. For seawater strains, the f/2 culture medium was used [50,51], while for freshwater cultures, BG11 medium was applied [52,53], except for *E. cantabrica*, which required BBM + vit + 2N [54,55]. The two species of *A. platensis* tested were cultured using modified Spirulina Medium (SpM) [56] (Table 1). Cultures were started at an optical density (OD) of 0.1 (Shimadzu UV-1900 UV-VIS spectrophotometer, Duisburg, Germany) at 680 nm and allowed to grow until the beginning of the stationary phase. At this point, unicellular cultures were harvested by centrifugation (Beckmann Coulter Avanti JXN-26 centrifuge, Brea, CA, USA) at 10,000 rpm for 20 min and filamentous strains were filtrated through a nylon 100 µm mesh size. Temperature, OD, and pH were measured daily, excluding weekends. The specific growth rate in the exponential growth phase (μ_exp_, which is the slope of the growth rate curve in the exponential phase) was calculated according to Equation (1):

μ_exp_ = l*n*(α2/α1)/(t2 − t1), (1)

where α1 and α2 are the absorbance readings at the beginning and at the end of exponential growth phase, at times 1 (t1) and 2 (t2), respectively. Productivity was calculated as the rate of the daily biomass increase during the exponential phase in g cell dry weight (CDW) l^−1^ d^−1^. The biomass of each strain was harvested at the stationary phase by centrifugation and then the wet biomass was freeze-dried and stored at −20 °C until extraction. Dry biomass (100 mg) of each strain was used for ash content determination by heating at 600 °C for 6 h in a muffle furnace. The remaining ash was slowly cooled down in a desiccator and its mass used to calculate the ash content percentage (% DW) (Figure S1, Supplementary Material). As expected, marine microalgae exhibited higher ash content compared to the freshwater strains. The ash content was considered for the calculation of all remaining chemical composition.

### 3.3. Total Phenol Content and Antioxidant Activity

Phenols extracts were obtained by mixing 10 mg of lyophilized biomass with 1 mL of ethanol, small glass beads were added to the tubes, and the mixture was homogenized using a laboratory mixer mill (5 min at 30 Hz, Retsch MM400, Haan, Germany). Then, the solution was left overnight under agitation (1200 rpm), centrifuged at 13,000 rpm for 10 min. Extractions were performed in triplicate. TPC was determined by spectrophotometry according to the method described by Singleton and Rossi with some modifications [57]. Briefly, 100 μL of extract was mixed with 1100 μL of distilled water and 75 μL of Folin–Ciocalteu reagent, then 225 μL of sodium carbonate solution (20%, *w*/*v*) was added to the mixture. The test tubes were then allowed to stand in the dark for 2 h at room temperature. The absorbance was read at 760 nm (Shimadzu UV-1900i UV-Vis Spectrophotometer, Duisburg, Germany) and TPC was expressed in terms of gallic acid equivalents for gram of dry weight (mg GAE g^−1^ DW). A calibration curve ranging from 10 to 200 μg∙mL^−1^ (R^2^ = 0.9962) was used to quantify the TPC in the algae extracts.

The scavenging activity of the 20 algae extracts was evaluated according to Blois with some modifications [58]. Briefly, a stock solution of DPPH 0.2 mM was prepared in methanol (90% *v*/*v*). The stock solution of DPPH was diluted in methanol 90% until a final OD (optical density) reached a value between 0.8 and 0.955 nm. A total of 1 mL of the diluted DPPH solution was mixed with 40 µL of algae extract and the mixture was left in dark for 30 min. A Trolox standard curve was plotted with a range 0.01–2 mM (R^2^ = 0.9981). The absorbance was read at 517 nm and results were expressed as mmol∙L^−1^ of Trolox equivalent for gram of dry weight (mmol∙L^−1^ TE g^−1^ DW).

### 3.4. Pigment Analysis

Determination of pigments content was carried out spectrophotometrically according to Lichtenthaler and Buschmann method [59]. Algae extracts were prepared as described in the previous section and the absorbance of samples was measured at 470, 648.6, and 664.1 nm. The content of chlorophyll *a*, chlorophyll *b*, and total carotenoids were calculated using the Lichtenthaler equations previously reported [59]:

$${Chl}_{a}\;\left(µg/mL\right)=13.36\times A_{664.1}-5.19\times A_{648.6}$$

$${Chl}_{b}\;\left(µg/mL\right)=27.43\times A_{648.6}-8.12\times A_{664.1}$$

$${Car}_{total}\;\left(µg/mL\right)=(1000\times A_{470}-2.13\times {Chl}_{a}-97.64\times {Chl}_{b})∕209$$

For HPLC analysis, pigment profiles were identified according to the method described by Zapata et al. [60]. Fresh algal cultures (5 mL) were filtered through glass fiber filters (diameter: 47 mm, pore size: 0.7 μm) and stored in glass tubes at −80 °C until further extraction. Then, pigments were extracted from the frozen filters with 5 mL of acetone (90% *w*/*v*) in a Teflon-lined screw-capped test tubes and allowed to stand 10 min in the ice. The mixture was vortexed and sonicated in a sonicator bath for 5 min and then filtered into a HPLC amber vial through a 0.22 μm filter. All steps were performed in ice and in a dark room, using a green light to avoid pigments degradation. Extractions were performed in triplicate. Pigments analysis was carried out using an HPLC system (Waters Alliance 2695, Milford, MA, USA), coupled with a Photodiode Array Detector (PAD, Waters 2996, Milford, MA, USA) operating between 350 and 750 nm according to the method described by Zapata et al. [60]. Samples were injected in a Symmetry C8 column (4.6 × 150 mm × 3.5 µm) kept at 25 °C. The mobile phase was (A) methanol; (B) acetonitrile; (C) 0.025 M pyridine, and (D) acetone. The gradient was the following: 0–22 min 50%A, 25%B, 25%C; 22–28 min 38%A, 39%B, 15%C; 28–38 min 21.4%A, 58.3%B, 1.3%C; 38–40 min 50%A, 25%B, 25%C. The flow rate was fixed at 1 mL∙min^−1^. Chromatograms were extracted at 450 nm and pigments were identified by comparing their retention time and visible spectra to those of the authentic standards. When the authentic standards were not available pigments were putatively identified comparing their RT and UV spectra with the related standards reported in the literature [25]. The blue:red ratio for chlorophylls and the band ratio for carotenoids were calculated from the absorption spectra.

Absolute quantification of lutein and fucoxanthin was determined for all microalgae and cyanobacteria using external calibration curves of pure analytical standards ranging from 0.05 to 5 µg∙mL^−1^ (R^2^ = 0.999). The limit of detection (LOD) and limit of quantification (LOQ) were 0.05 and 0.13 µg∙mL^−1^ for fucoxanthin and 0.04 and 0.11 µg∙mL^−1^ for lutein, respectively. In the absence of pure analytical standards, other pigments were relatively quantified, and their presence is reported as % of the total pigments.

### 3.5. Lipids Extraction and Fatty Acids Profile by GC-FID

Total lipids (TL) were extracted according to the method described by Bligh and Dyer with small modifications [61]. Briefly, 300 mg of freeze-dried biomass was homogenized in 10 mL of chloroform/methanol (2:1, *v*/*v*) with 0.01% BHT (*w*/*v*) as an antioxidant for 5 min using an ice bath to maintain a low temperature (Polytron, PT 2100, Lucerne, Switzerland). Then, 2.5 mL of KCl 0.88% was added to the mixture and the tubes were vortexed for 1 min and centrifuged at 2000 rpm for 5 min at 4 °C (Beckman coulter Avanti JXN-26, Brea, CA, USA). The upper phase was discarded while the organic phase was filtered, and a second extraction was performed on the interphase by adding 3 mL of chloroform/methanol (2:1), vortexed for 1 min, and centrifuged. The organic extract was filtered and combined with the previous extract. TL were gravimetrically determined after evaporation of the organic solvent under a stream of nitrogen, and they are reported as % of dry biomass. TL were stored at −80 °C in 10 mg∙mL^−1^ chloroform/methanol (2:1, *v*/*v*) under an inert atmosphere of nitrogen to prevent oxidation. Lipids extractions were performed in triplicate.

Fatty acids methyl esters (FAMEs) were obtained by acid-catalyzed transmethylation of dry lipids extracts using 2 mL of 1% sulphuric acid in methanol and 1 mL of toluene to improve transmethylation of non-polar lipids [62]. To avoid oxidation the tubes were filled with N_2_ gas, then the reaction was performed at 50 °C for 16 h. After the transmethylation, the tubes were left at room temperature to cool down, then 4 mL of hexane:diethyl ether (1:1 *v*/*v*) with BHT (0.01% *w*/*v*) and 2 mL of KHCO_3_ (2% *w*/*v*) were added to each tube. The tubes were vortexed, and the hexane phase was recovered after centrifugation at 2000 rpm for 5 min at 4 °C. The aqueous phase was washed with 4 mL of hexane:diethyl etere, and after centrifugation the hexane phase was collected and combined to the previous organic phase to be dried under a stream of inert nitrogen. FAMEs were resuspended in hexane and filtered through a 0.45 µm filter prior injection. FAMEs were analyzed using a gas chromatograph equipped with a flame ionization detector (PerkinElmer Clarus 690 GC-FID, Shelton, CT, USA) and a fused silica capillary column Elite-WAX (30 m × 0.32 mm × 0.5 µm, PerkinElmer, Shelton, CT, USA). Helium was used as carrier gas and column flow was held at 1.5 mL∙min^−1^. Samples (1 μL) were injected in split mode (1:20) and the analysis parameters were: injector temperature at 240 °C and detector temperature at 250 °C. The initial column temperature was 50 °C for 1 min, before being increased to 150 °C at a rate of 40 °C min^−1^, then increased to 200 at 2 °C min^−1^, then to 214 at 1 °C min^−1^, then to 230 at 40 °C min^−1^, to a final temperature of 240 at 10 °C min^−1^ and hold for 10 min. Nitrogen gas was used as make up gas (30 mL min^−1^); flow of hydrogen gas and synthetic air were provided at 30 and at 450 mL∙min^−1^, respectively. FAMEs were identified by comparing their retention times with those of authentic standards available in our lab. Nonadecanoic acid (C19:0) was used as internal standard. The relative quantification of FAMEs was calculated as percentage of the total FAs presents in the analyzed microalgae.

### 3.6. Protein Analysis

The protein content was determined with Kjeldahl method (Foss Kjeltec 8100, Hillerød, Denmark) equipped with a digestor, a scrubber and a distillation unit, using 200 mg of lyophilized dry biomass. Samples were digested for 1 h at 420 °C with 12 mL of sulfuric acid and 2 tablets of catalyst (Kjeltabs Foss). After digestion, samples were cooled and distilled with a distillation unit. In the collection flask, 30 mL of boric acid 4% and 8 drops of a mixed color indicator were added for the final titration carried out with 0.1 N hydrochloric acid. An N-to-protein conversion factor of 4.78 was used to calculate total protein from total nitrogen [63]. Ammonium sulphate and acetanilide were used as standards in the distillation and digestion processes, respectively. Analyses were performed in triplicate.

### 3.7. Preparation of Crude Extract for Antibacterial Assay

Lyophilized dry biomass (500 mg) was extracted according to the method described by de Falco et al. (2017) with some modification [64]. Briefly, 20 mL of a biphasic solvent mixture composed of cold dichloromethane:methanol:water (2:1:1) was added to dry biomass and vortexed. To ensure efficient lysis of cell membranes and to promote the escape of all metabolites, the mixture was sonicated for 15 min with an ultrasonic homogenizer 4710 at 20 kHz with a power output of 27 watts. During the extraction, samples were kept in ice to keep the temperature below 30 °C. After centrifugation at 6000 rpm for 10 min at 4 °C (Sorvall Legend XT/XTR, Thermo Scientific, Waltham, MA, USA) the aqueous and organic fractions were accurately separated and filtered, with particular attention to discard the interphase. The extraction and subsequent centrifugation were repeated two more time on the interphase, and the collected extracts were evaporated to dryness under vacuum (Rotavapor Buchi, R-300, Flawil, Switzerland). The dried samples were stored at 4 ◦C until further analysis. All extractions were performed in triplicate. The organic extracts were reconstituted in Dimethyl sulfoxide (DMSO) to have a concentration of 2048 µg∙mL^−1^.

### 3.8. Assesment of Antimicrobial Activity: Determination of Minimum Inhibitory Concentration and Minimum Bactericidal Concentration

Twenty strains *of Staphylococcus aureus* were used for the study (Table S2 of Supplementary Material). Before each antimicrobial susceptibility test, the strains were incubated in Trypticase Soy Agar with 5% Sheep Blood (Becton Dickinson GmbH, Heidelberg, Germany) at 37 °C for 24 h aerobically.

The MIC of bioactive compounds derived from microalgae and cyanobacteria is defined as the lowest concentration that inhibits the growth of the bacteria. We determined the MICs using the broth microdilution method [65]. Mueller–Hinton broth (MHB) (Becton Dickinson, Sparks, MD, USA) or MHB supplemented with DMSO (25%) were used to determine the MICs for aqueous or organic fractions, respectively. Briefly, a two-fold concentrate of each medium (50 μL) was placed into wells of a 96-well polypropylene round bottom plate (Watson Bio Lab, Tokyo, Japan). To obtain the working stock solutions of the different seaweed extracts, the aqueous extracts were first diluted to the highest concentration (30,720 μg mL^−1^) in sterile 0.9% saline solution and the organic ones, were diluted to the highest concentration (2048 μg mL^−1^) in sterile 0.9% saline solution plus DMSO (25%). The stock solutions were filtered with a 0.22 µm sterile filter (Millex^®^-GS, Merck Millipore. Ltd., Cork, Ireland) to guarantee sterility. The sterility of the extracts was verified by inoculating 50 µL of each extract on Trypticase Soy Agar with 5% Sheep Blood (Becton Dickinson GmbH, Heidelberg, Germany) at 37 °C for 24 h aerobically. Serial two-fold dilutions of each algae extract were made in a concentration range from 1024 to 0.5 μg mL^−1^. Then, overnight bacterial cultures (37 °C with shaking at 120 rpm) were diluted to 5 × 10^6^ colony-forming units (cfu)/mL with sterile phosphate-buffered saline (PBS), and 10 μL was inoculated into each well. Finally, the plates were incubated at 37 °C for 22 h, after which the MICs were determined. The MIC was determined as the lowest concentration with no visible growth. For MBC tests, 100 μL aliquots from each incubated sample for MIC test were spread onto Müller–Hinton agar plates and subsequently incubated for 20 h at 37 °C. The MBC was determined as the lowest concentration at which no visible bacterial growth was observed on the agar. All MIC and MBC tests were performed three times, independently. Finally, MIC50 and MIC90 were defined as the minimum inhibitory concentration needed to inhibit the growth of 50% and 90% of the *S. aureus* strains used, respectively.

### 3.9. Statistical Analysis

All analyses were performed in triplicates for each microalgae sample. Results are reported as mean ± standard deviation of replicates (*n* = 3). Analysis of variance (One-Way ANOVA, *p* < 0.05) and the correlation between antioxidant activity and total carotenoids or total phenolic content were assessed using IBM SPSS Statistic 24. Multivariate data analysis was performed using SIMCA software (version 16, Umetrics, Umeå, Sweden). The dataset was mean-centered and Pareto scaled.

## 4. Conclusions

While microalgae have demonstrated great potential as a source of numerous beneficial compounds for several diverse applications, only a limited number of species or derivatives have received approval for human consumption. Among the strains analyzed in this study, *E. cantabrica*, *C. giraudii*, and *H. pluvialis* emerged as the most interesting due to their high phenol content and outstanding antioxidant activity, suggesting their potential to reduce oxidative stress. It would be interesting to evaluate their anti-inflammatory properties as well and to study these activities in *E. cantabrica* also when it is not in the palmella phase. Furthermore, *H. rubescens*, *Picochlorum* sp., and *H. pluvialis* also deserve particular attention for human consumption due to their low content of SFAs and n6:n3 ratio. The rapid growth rates of *A. minutissima* and *C. stagnale* combined with their high protein content, make them promising candidates for sustainable, protein-rich supplements. Regarding the organic extracts, *Entomoneis* sp., *C. giraudii*, *I. galbana*, *Picochlorum* sp., *C. stagnale*, and *Nodularia* sp., presented the highest antimicrobial effects. Additionally, GC-FID analysis combined with multivariate statistical approaches demonstrated that fatty acids could serve as biomarkers for elucidating chemotaxonomic relationships among various microalgal species. The results obtained from this study give interesting insights on the potential use of selected microalgae strains, given their health benefits, for future industrial application from an intensive production perspective. While it may seem evident that genetically distinct groups lead to distinct biochemical profiles, emphasizing the intricate relationship between genetic diversity, metabolic pathways, and the resulting array of valuable metabolites can serve as a compelling incentive for deeper exploration of this diversity, enabling more targeted selection of strains, species, and genera for specific biotechnological applications. Additional studies on other strains of the same species are necessary to confirm their relevance in the pharmaceutical and nutraceutical sectors.

## Figures and Tables

**Table 1:** List of microalgae strains selected for this study, including their geographical origin, culture media, and the number of data points (*n*) used to calculate growth rate (µ) and biomass productivity.

Species	BEA Code	Division (Class)	Geographical Origin	Culture Medium	*n*	µ (d^−1^)	Biomass Productivity (gCDW·l^−1^·d^−1^)
EUKARYOTES							
*Chrysoreinhardia giraudii*	BEA 0313B	Heterokontophyta (Pelagophyceae)	Spain, Canary Islands, Gran Canaria. La Aldea harbor	F/2	12	0.277 ± 0.054	0.159 ± 0.031
*Chlorella vulgaris*	BEA 0045B	Chlorophyta (Trebouxiophyceae)	Cape Verde, Maio. Porto Ingles saltwork	F/2	16	0.414 ± 0.100	0.030 ± 0.007
*Entomoneis* sp.	BEA 0505B	Heterokontophyta (Bacillariophyceae)	Spain, Canary Islands, La Palma. Volcano new beach, squeezed from *Ulva* sp.	F/2	12	0.362 ± 0.027	0.059 ± 0.012
*Euglena cantabrica*	BEA 0937B	Euglenophyta (Euglenophyceae)	Spain, Canary Islands, Gran Canaria. Maspalomas coastal lagoon	BBM + vit + 2N	8	0.109 ± 0.011	0.049 ± 0.008
*Haematococcus pluvialis*	BEA 1360B	Chlorophyta (Chlorophyceae)	Spain, Canary Islands, Gran Canaria. Agaete rock pool	BG11	16	0.364 ± 0.105	0.023 ± 0.004
*Hallochlorella rubescens*	BEA 0069B	Chlorophyta Chlorophyceae)	Spain, Canary Islands, Fuerteventura. Lobos island sandy crust	F/2	8	0.249 ± 0.050	0.052 ± 0.018
*Isochrysis galbana*	BEA 1751B	Haptophyta (Cocolithophyceae)	Spain, Canary Islands, Gran Canaria. Bocacangrejo saltwork	F/2	20	0.329 ± 0.025	0.033 ± 0.026
*Nannochloropsis gaditana*	BEA 1883B	Heterokontophyta (Eustigmatophyceae)	Spain, Canary Islands, Gran Canaria. Taliarte coast, marine water sample	F/2	20	0.347 ± 0.025	0.021 ± 0.004
*Picochlorum* sp.	BEA 0154B	Chlorophyta (Trebouxiophyceae)	Spain, Canary Islands, Gran Canaria. Maspalomas coastal lagoon	F/2	24	0.350 ± 0.047	0.017 ± 0.004
*Rhodosorus marinus*	BEA 1286B	Rhodophyta (Stylonematophyceae)	Spain, Canary Islands, Gran Canaria. Taliarte rock pool	F/2	8	0.444 ± 0.074	0.112 ± 0.019
PROKARYOTES							
*Anabaena minutissima*	BEA 0300B	Cyanophyta (Cyanobacteria)	Spain, Canary Islands, Fuerteventura. Ajuy freshwater upwelling on the coast	BG11	12	0.473 ± 0.032	0.041 ± 0.010
*Arthrospira platensis*	BEA 0007B	Cyanophyta (Cyanobacteria)	Chad, Lake Chad	SpM	8	0.329 ± 0.046	0.064 ± 0.004
*Arthrospira platensis*	BEA 1257B	Cyanophyta (Cyanobacteria)	Spain, Canary Islands, Fuerteventura. Los Molinos dam	SpM	16	0.309 ± 0.037	0.040 ± 0.012
*Cylindrospermum stagnale*	BEA 0605B	Cyanophyta (Cyanobacteria)	Spain, Canary Islands, La Gomera. Garajonay. National Park, slimy material from a wet rock	BG11	24	0.452 ± 0.212	0.013 ± 0.005
*Microcystis aeruginosa*	BEA 0699B	Cyanophyta (Cyanobacteria)	Spain, Canary Islands, Tenerife. La Orotava Botanical Garden Pond	BG11	20	0.346 ± 0.046	0.017 ± 0.005
*Nodularia* sp.	BEA 0866B	Cyanophyta (Cyanobacteria)	Spain, Canary Islands, Gran Canaria. Marine water sample.	F/2	16	0.274 ± 0.097	0.062 ± 0.021
*Nostoc commune*	BEA 0024B	Cyanophyta (Cyanobacteria)	Spain, Canary Islands, Gran Canaria. On a trunk of *Phoenix canariensis*	BG11	20	0.475 ± 0.129	0.024 ± 0.009
*Nostoc* sp.	BEA 0864B	Cyanophyta (Cyanobacteria)	Morocco, Rabat-Salè-Zemmour-Zaër, Rabat. Marine water sample	BG11	24	0.388 ± 0.060	0.029 ± 0.017
*Synechococcus elongatus*	BEA 1031B	Cyanophyta (Cyanobacteria)	Spain, Canary Islands, Gran Canaria. Ayagaures dam, squeezed from *Nitella* sp.	BG11	16	0.212 ± 0.060	0.021 ± 0.006
*Synechocystis* sp.	BEA 1833B	Cyanophyta (Cyanobacteria)	Spain, Toledo. Tirez Lagoon-Villacañas, hypersaline lagoon	BG11	12	0.360 ± 0.042	0.024 ± 0.008

**Table 2:** Identified pigments with their related RT (min), λ_max_ (nm) and band ratio.

Identified Pigments (Abbreviation)	RT (Min)	λ_max_ (nm)	Band Ratio
Chlorophyll-*c_3_* (Chl-*c_3_*) *	9.22	458	28 ^a^
Chlorophyll-*c_2_* (Chl-*c_2_*) *	13.31	453; 585; 631	9 ^a^
Chlorophyll-*c_1_* (Chl-*c_1_*)	14.18	448; 581; 633	7 ^a^
Fucoxanthin (Fuco) *	21.18	450	-
Neoxanthin (Neo) *	21.20	415; 442; 468	54 ^b^
Violaxanthin (Viola) *	22.14	415; 438; 467	91 ^b^
Synechoxanthin (Syne)	23.21	453; 479	35 ^b^
Myxol quinovoside (Myxo)	24.59	452; 477; 508	57 ^b^
Diadinoxanthin (Diadino) *	25.63	424; 448; 477	70 ^b^
Caloxanthin (Cal)	26.34	452; 478	44 ^b^
Zeaxanthin (Zea) *	28.10	454; 480	34 ^b^
Lutein (Lut) *	28.37	423; 447; 475	62 ^b^
Canthaxanthin (Cantha)	29.56	475	-
Chlorophyll-*b* (Chl-*b*) *	32.03	464; 654	2 ^a^
Chlorophyll-*a* allomer 1 (Chl-*a* allo 1)	32.89	419;657	0.87 ^a^
Chlorophyll-*a* allomer 2 (Chl-*a* allo 2)	33.04	430; 660	0.88 ^a^
Chlorophyll-*a* (Chl-*a*) *	33.49	431; 660	0.85 ^a^
Chlorophyll-*a* epimer (Chl-*a’*) *	33.81	431; 660	0.57 ^a^
Pheophytin-*b* (Phe-*b*)	34.63	437; 534; 656	2.6 ^a^
*β,β*-Carotene (*β,β*-Car)	36.20	455; 482	26 ^b^

^a^ blue:red ratio. ^b^ % III:II. * Pigments identified with analytical standards.

**Table 3:** Absolute quantification of fucoxanthin and lutein expressed in mg g^−1^ of dry weight (DW). Results are reported as mean ± standard deviation (*n* = 3).

Species	Code	Fucoxanthin mg g^−1^ DW	Lutein mg g^−1^ DW
*Chrysoreinhardia giraudii*	BEA 0313B	1.46 ± 0.08	-
*Chlorella vulgaris*	BEA 0045B	-	0.25 ± 0.04
*Entomoneis* sp.	BEA 0505B	4.35 ± 0.05	-
*Haematococcus pluvialis*	BEA 1360B	-	2.96 ± 0.17
*Hallochlorella rubescens*	BEA 0069B	-	<LOQ
*Isochrysis galbana*	BEA 1751B	0.54 ± 0.06	-
*Nannochloropsis gaditana*	BEA 1883B	-	<LOQ
*Nostoc commune*	BEA 0024B	<LOQ	-
*Picochlorum* sp.	BEA 0154B	-	0.51 ± 0.04

**Table 4:** Total lipids (TL), saturated fatty acids (SFAs), monounsaturated fatty acids (MUFAs), polyunsaturated fatty acids (PUFAs), n6:n3 ratio and protein content are reported. Different case letters indicate significant differences (*p* < 0.05) as determined by the post hoc Tukey’s test.

	TL	ΣSFAs	ΣMUFAs	ΣPUFAs	Σn6/Σn3	Protein
Eukaryote						
*C. giraudii*	11.3 ± 0.04	35.2 ± 0.53	6.0 ± 0.19	36.9 ± 0.60	0.2 ± 0.001	13.9 ± 0.18 g
*C. vulgaris*	18.5 ± 0.05	34.2 ± 0.54	30.7 ± 0.58	24.7 ± 0.78	1.5 ± 0.05	19.8 ± 0.18 f
*Entomoneis* sp.	12.0 ± 0.004	42.1 ± 0.12	34.1 ± 0.04	12.6 ± 0.07	0.2 ± 0.004	19.5 ± 0.01 f
*E. cantabrica*	20.2 ± 0.02	14.6 ± 0.06	4.0 ± 0.15	55.1 ± 0.11	1.6 ± 0.01	38.9 ± 0.23 c
*H. pluvialis*	18.9 ± 0.04	16.2 ± 0.69	6.8 ± 0.22	36.1 ± 1.38	0.5 ± 0.04	44.2 ± 0.08 b
*H. rubescens*	12.9 ± 0.04	23.4 ± 0.10	33.0 ± 0.11	28.7 ± 0.05	0.4 ± 0.004	18.1 ± 0.43 fg
*I. galbana*	14.4 ± 0.01	47.7 ± 0.32	25.5 ± 0.27	18.8 ± 0.54	0.4 ± 0.002	28.3 ± 0.95 e
*N. gaditana*	18.9 ± 0.03	35.1 ± 0.25	31.0 ± 0.35	26.8 ± 0.46	0.4 ± 0.003	31.2 ± 0.06 e
*Picochlorum* sp.	15.6 ± 0.03	25.3 ± 0.14	8.6 ± 0.11	35.3 ± 0.14	1.0 ± 0.02	37.8 ± 1.18 cd
*R. marinus*	4.3 ± 0.001	49.2 ± 0.23	20.5 ± 0.18	25.2 ± 0.08	0.3 ± 0.02	9.2 ± 0.37 h
Cyanobacteria						
*A. minutissima*	12.9 ± 0.05	34.0 ± 2.33	20.0 ± 1.76	33.1 ± 4.60	0.3 ± 0.05	54.9 ± 0.14 a
*A. platensis*	10.6 ± 0.01	42.5 ± 0.36	7.7 ± 0.43	37.8 ± 0.18	61.5 ± 3.76	44.2 ± 0.46 b
A. platensis*	7.1 ± 0.03	41.3 ± 1.08	12.4 ± 0.12	34.5 ± 0.33	31.7 ± 11.4	44.2 ± 0.09 b
*C. stagnale*	13.2 ± 0.05	28.4 ± 1.20	8.2 ± 0.13	39.5 ± 1.54	0.7 ± 0.07	55.4 ± 0.46 a
*M. aeruginosa*	8.6 ± 0.01	57.2 ± 0.19	5.6 ± 0.44	25.3 ± 0.37	2.5 ± 0.05	57.6 ± 0.51 a
*Nodularia* sp.	7.5 ± 0.002	38.8 ± 0.46	26.0 ± 0.46	22.6 ± 0.62	1.0 ± 0.01	34.1 ± 0.13 d
*N. commune*	4.4 ± 0.01	29.6 ± 0.45	23.8 ± 0.16	33.9 ± 0.49	0.9 ± 0.02	19.4 ± 5.64 f
*Nostoc* sp.	14.5 ± 0.05	34.6 ± 0.33	18.5 ± 0.20	36.8 ± 0.54	0.8 ± 0.02	55.7 ± 1.26 a
*S. elongatus*	5.7 ± 0.02	44.5 ± 0.14	31.5 ± 0.19	17.7 ± 0.44	13.5 ± 3.24	36.3 ± 0.80 cd
*Synechocystis* sp.	17.7 ± 0.04	34.5 ± 0.13	31.4 ± 0.19	27.3 ± 0.24	0.4 ± 0.003	28.8 ± 0.92 e

TL and protein contents are expressed as % of freeze-dried biomass. ΣSFA, ΣMUFA and ΣPUFA are expressed as % of total fatty acids. *A. platensis* refers to BEA 0007B strain. A. platensis* refers to BEA 1257B strain.

**Table 5:** MIC50 and MIC90 (µg/mL) values of the organic extracts from 10 eukaryotes and 10 prokaryotes. *A. platensis* refers to BEA 0007B strain. A. platensis* refers to BEA 1257B strain.

	MIC50	MIC90
	(µg/mL)	
Eukaryotes		
*C. giraudii*	128	128
*C. vulgaris*	1024	>1024
*Entomoneis* sp.	32	32
*E. cantabrica*	>1024	>1024
*H. pluvialis*	512	>1024
*H. rubescens*	>1024	>1024
*I. galbana*	128	256
*N. gaditana*	512	1024
*Picochlorum* sp.	128	256
*R. marinus*	128	512
Prokaryotes		
*A. minutissima*	>1024	>1024
*A. platensis*	1024	>1024
A. platensis*	1024	>1024
*C. stagnale*	128	256
*M. aeruginosa*	256	512
*Nodularia* sp.	128	512
*N. commune*	>1024	>1024
*Nostoc* sp.	512	1024
*S. elongatus*	>1024	>1024
*Synechocystis* sp.	256	512

## Data Availability

Dataset available on request from the authors.

## Supplementary Materials

The following supporting information can be downloaded at: https://www.mdpi.com/article/10.3390/md24050171/s1, Figure S1: Ash content in lyophilized dry biomass of 10 eukaryota and 10 cyanobacteria reported as % of dry weight. *A. platensis* refers to the species with code BEA0007B, *A. platensis** refers to the species with code BEA1257B. Figure S2: Identified pigments from 10 eukaryotic microalgae extracts. Values are reported as % of total identified pigments, and they represent the mean ± standard deviation (*n* = 3). Figure S3. Identified pigments from 10 cyanobacteria extracts. Values are reported as % of total identified pigments, and they represent the mean ± standard deviation (*n* = 3). Table S1: Fatty acids profile (detected as FAMEs) of the selected microalgae and cyanobacteria. Values are expressed as % of total fatty acids and presented as mean ± standard deviation (*n* = 3). Table S2: MIC and MBC results for each *S. aureus* strain against different organic microalgae extracts selected in this study. MIC50 and MIC90 values are presented at the end of the columns corresponding to each microalgae strain.
