# Supplementary material for: Bioactive Compounds from Microalgae and Cyanobacteria: Evaluation of Their Antioxidant and Antimicrobial Activities
Source: Mar Drugs. 2026 May 9;24(5):171. doi: 10.3390/md24050171 (PMC13208498; doi:10.3390/md24050171)
Supplement: Supplementary file 1 [file marinedrugs-24-00171-s001.zip › Supplementary material_Figures S1,S2 and S3.pdf]

Supplementary materials

Bioactive compounds from microalgae and cyanobacteria: evaluation of their antioxidant and antimicrobial activities

Bruna de Falco [1,2,] *, Carlos José Martel-Benítez [3,4], and Carlos Almeida [1], Attilio Anzano [2], Francesco Pisapia [1,5], Jose Luis Martin-Barrasa [3,4,6], Antera Martel Quintana [7] and Juan Luis Gómez Pinchetti [7]

1 Banco Español de Algas, Fundación Parque Científico Tecnológico, Universidad de Las Palmas de Gran Canaria, Muelle de Taliarte s/n, 35214 Telde, Las Palmas, Canary Islands, Spain; calmeida@marinebiotechnology.org
2 Department of Agricultural Sciences, University of Naples Federico II, Via Università 100, 80055, Portici, Italy; bruna.defalco@unina.it, attilio.anzano@unina.it
3 Fish Health and Infectious Diseases Group, University Institute of Animal Health and Food Safety (IUSA), University of Las Palmas de Gran Canaria, Carretera de Trasmontana s/n, 35416 Arucas, Spain; carlos.benitez@ulpgc.es, joseluis.martin@ulpgc.es
4 Research Unit Hospital Universitario de Gran Canaria, Dr. Negrín, Fundación Instituto de Investigación Sanitaria de Canarias (FIISC), Bco. de la Ballena s/n 35011 Las Palmas de Gran Canaria, Spain.
5 Faculty of Health Sciences, University Fernando Pessoa-Canarias, C/ la Juventud s/n, 35450 Santa María de Guía, Las Palmas, Canary Islands, Spain; fpisapia@ufpcanarias.es
6 CIBER de Enfermedades Infecciosas (CIBERINFEC), Instituto de Salud Carlos III, Madrid, Spain
7 Banco Español de Algas, Instituto de Oceanografía y Cambio Global (IOCAG), Universidad de Las Palmas de Gran Canaria, 35214, Telde, Las Palmas, Canary Islands, Spain; juan.gomez@ulpgc.es, amartel@marinebiotechnology.org
* Corresponding author: bruna.defalco@unina.it

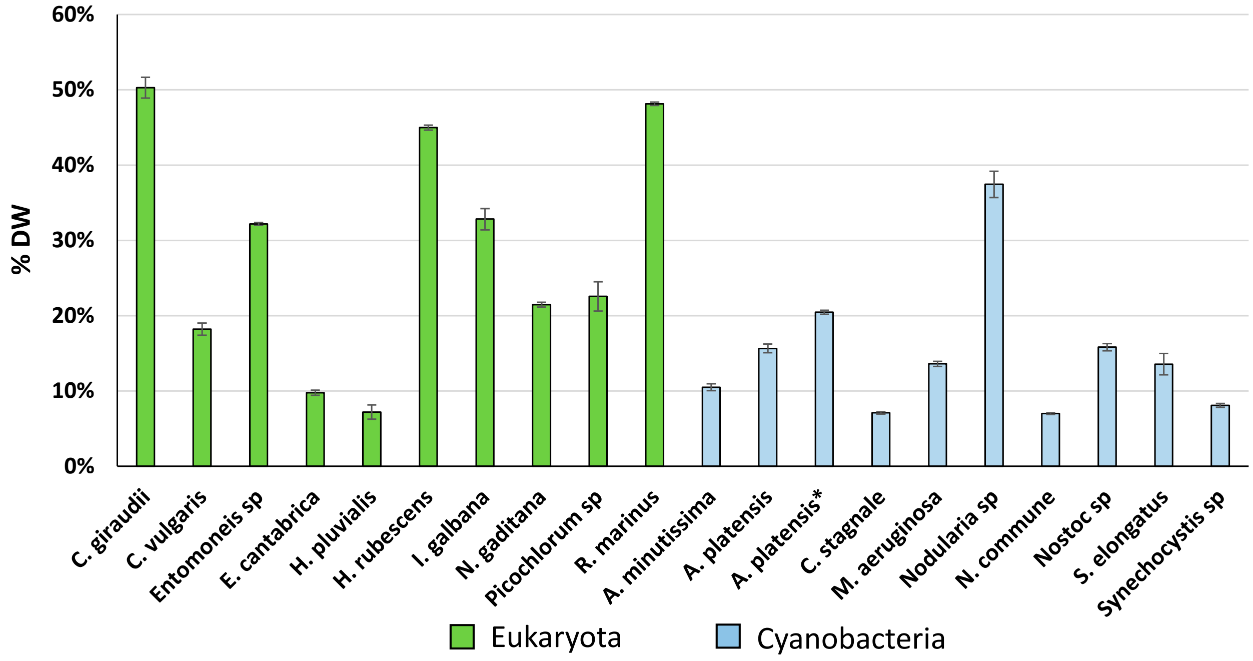

Figure S1. Ash content in lyophilized dry biomass of 10 eukaryota and 10 cyanobacteria reported as % of dry weight (% DW). *A. platensis* refers to the species with code BEA0007B, *A. platensis** refers to the species with code BEA1257B.

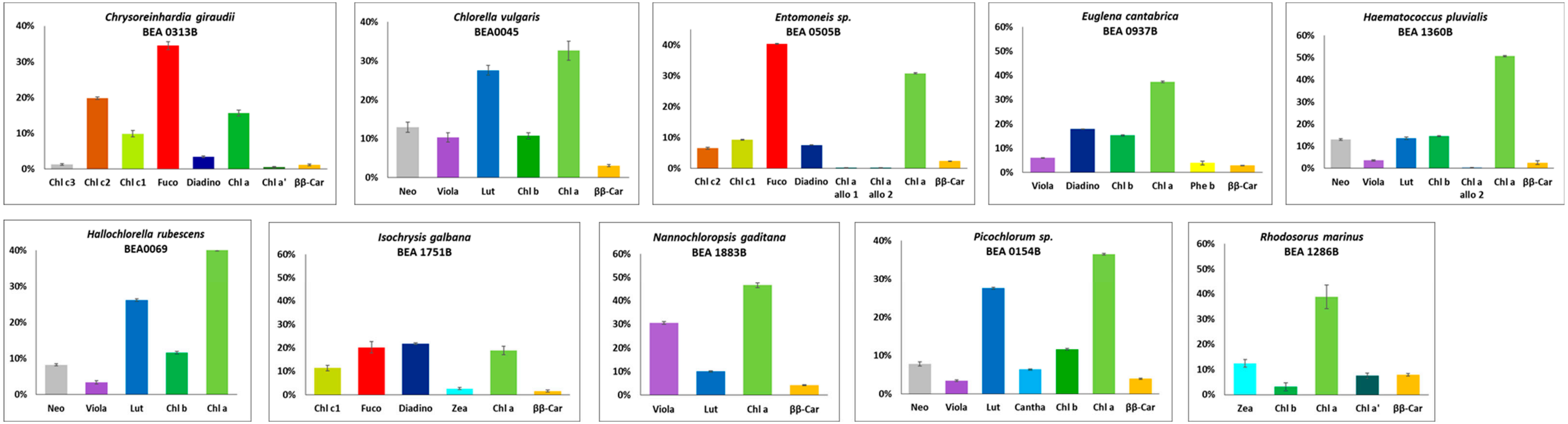

Figure S2. Identified pigments from 10 eukaryotic microalgae extracts. Values are reported as % of total pigments and they represent the mean ± standard deviation (n = 3).

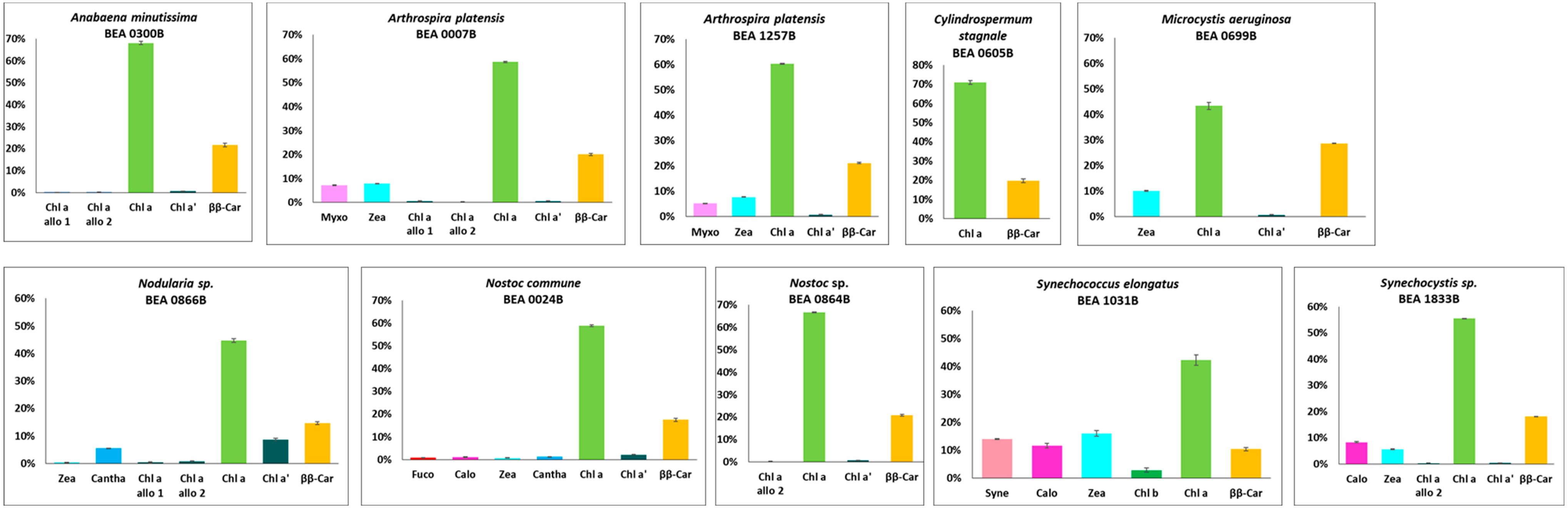

Figure S3. Identified pigments from 10 cyanobacteria extracts. Values are reported as % of total pigments and they represent the mean ± standard deviation (n = 3).
